# Incidence of Sindbis Virus in Hospitalized Patients With Acute Fevers of Unknown Cause in South Africa, 2019–2020

**DOI:** 10.3389/fmicb.2021.798810

**Published:** 2022-02-07

**Authors:** Kgothatso Meno, Clarence Yah, Adriano Mendes, Marietjie Venter

**Affiliations:** Zoonotic Arbo and Respiratory Virus Program, Department of Medical Virology, Centre for Viral Zoonoses, University of Pretoria, Pretoria, South Africa

**Keywords:** Sindbis virus, IgM, South Africa, acute febrile illness, neurological symptoms, indirect immunofluorescence assay, micro neutralization assay, hospitalized patients

## Abstract

**Background:**

Sindbis virus (SINV) is a mosquito-borne alphavirus that is widely distributed worldwide. Little is known about the febrile and neurological disease burden due to SINV in South Africa.

**Patients and Methods:**

Clinical samples of patients with acute febrile disease of unknown cause (AFDUC) were collected through the African Network for Improved Diagnostics, Epidemiology and Management of Common Infectious Agents at three sentinel hospital surveillance sites in South Africa. In total, 639 patients were screened using a PCR-based macroarray that can simultaneously detect nucleic acids of 30 pathogens, including SINV, from January 2019 to December 2020. Serum samples were randomly selected from the arbovirus season (January–June) and also screened with a commercial indirect immunofluorescence assay for anti-SINV IgM. In addition, 31 paired cerebrospinal fluid (CSF) specimens from the same patients were screened for IgM. Micro-neutralization assays were performed on all IgM-positive samples.

**Results:**

None of the specimens tested positive for SINV by molecular screening; however, 38/197 (19.0%) samples were positive for SINV-specific IgM. A total of 25/38 (65.8%) IgM-positive samples tested positive for SINV-neutralizing antibodies, giving an overall incidence of 12.7%. Furthermore, 2/31 (6.5%) CSF specimens tested positive for IgM but were negative for neutralizing antibodies. There was a higher incidence of SINV-positive cases in Mpumalanga (26.0%) than Gauteng province (15.0%). The most significant months for IgM-positive cases were April 2019 (OR = 2.9, *p* < 0.05), and May 2020 (OR = 7.7, *p* < 0.05).

**Conclusion:**

SINV or a closely related virus contributed to 12.7% of AFDUC cases in hospitalized patients during the late summer and autumn months in South Africa and was significantly associated with arthralgia, meningitis, and headaches.

## Introduction

Sindbis virus (SINV) is an arthropod-borne virus (arbovirus) within the *Alphavirus* genus in the family *Togaviridae* (Strauss and Strauss, 1994). SINV was first isolated from *Culex* mosquitoes in the Sindbis district, north of Cairo, Egypt, in the year 1952 (strain AR-339) (Taylor et al., 1955). Its transmission cycle is primarily enzootic circulating between birds and mosquitoes (Ling et al., 2019). Spillover from mosquitoes or birds into humans results in sporadic infections (Ling et al., 2019). SINV is considered an Old World alphavirus, and these are primarily arthritogenic (Adouchief et al., 2016). New World alphaviruses, such as Venezuelan, Eastern, and Western equine encephalitis virus, are considered more neuro-invasive (Go et al., 2014) for humans and horses although neurological SINV and Middelburg virus (MIDV) infections have been reported in horses in South Africa (SA) (van Niekerk et al., 2015).

SINV was first identified as a human pathogen in 1961 when it was first isolated in patients with fever in Uganda (Woodall et al., 1962) and has since been demonstrated to be the causative agent of a febrile illness associated with maculopapular rash, fatigue, and joint pain in humans in Africa, Europe, Australia, and Asia (McIntosh et al., 1976; Jupp and Nk, 1986; Strauss and Strauss, 1994; Laine et al., 2000, 2004; Brummer-Korvenkontio et al., 2002; Storm et al., 2014a,b; Berger, 2018). SINV is reported to affect all age groups with similar incidence between sexes (Espmark and Niklasson, 1984; Laine et al., 2000, 2004; Storm et al., 2014a; Berger, 2018). Sporadic cases of SINV infections, presenting with mild symptoms, are reported during the late summer and autumn months (Storm et al., 2014a,b). Previously, SINV infection was not considered a major public health concern in humans until epidemics in SA in April 1974 and 1983–1984 and in Finland, Sweden, and Russia in the 1980s (McIntosh et al., 1976; Jupp and Nk, 1986). The epidemic in SA in 1974 occurred after heavy rains and resulted in 4,000 cases of fever, myalgia, rash, and arthralgia. In 1981, there were 300 and 200 laboratory confirmed cases reported in Finland and the Soviet Union, respectively (Lvov et al., 1984; Niklasson, 1994; Brummer-Korvenkontio et al., 2002). The infections reported in SAa in the 1960s were especially prevalent in the Highveld region of the Transvaal and Orange Free State and along the Orange River, where high temperatures and irrigation create favorable conditions for the arthropod vector (Mcintosh et al., 1964; Laine et al., 2004). Sporadic cases and occasional outbreaks are reported by the National Institute for Communicable diseases in SA,^1^ but the burden of disease is not known, and cases are thought to be underreported.

The symptoms of SINV infections vary from self-limiting (2–21 days) to several years of illness, often associated with arthralgia (Kiwanuka et al., 1999; Laine et al., 2004). SINV usually causes acute febrile illness that may explain the underlying factors contributing to underreporting the disease (Tong et al., 2001; Laine et al., 2004; Storm et al., 2014a). Moreover, acute febrile illnesses are associated with high morbidity rates and mortality worldwide; however, the etiological agent is often not identified. Laboratory diagnosis of alphaviruses is performed using molecular tools, such as RT-PCR, but due to its short viremia, it is primarily dependent on serological assays that detect IgM and IgG antibodies. These include enzyme immunoassays, hemagglutination inhibition test, indirect immunofluorescence (IFA) or immunoblot assays. Despite, high levels of cross-reaction between alphaviruses using serum neutralization assays, they are used for confirmation (Clarke and Casals, 1958; Kurkela et al., 2008; Manni et al., 2008).

This study aimed to investigate if SINV contributed to acute febrile disease of unknown cause (AFDUC) with or without neurological signs in hospitalized patients of all age groups in the Highveld (Gauteng province) and the Lowveld (Mpumalanga province) of SA as well as the risk factors associated with SINV infections.

## Materials and Methods

### Ethical Approval

Ethical approval to conduct this study was granted by the University of Pretoria, Faculty of Health Sciences Research Ethics Committee Protocols 100/2017 (ANDEMIA study) and 101/2017 (National Health Laboratory Services, Virology Laboratory), 842/2019 and HUM017/1119 (MSc project ethics approval). Hospital approvals were obtained from Kalafong Hospital KPTH 33/2017 and the Department of Health Mpumalanga province.

### Study Design

Three hospitals were selected in the Gauteng and Mpumalanga provinces, SA, as sentinel sites for surveillance of AFDUC as part of the African Network for improved Diagnostics, Epidemiology and Management of common infectious Agents (ANDEMIA). These hospitals include Kalafong Tertiary Academic Hospital (Gauteng Province), Mapulaneng Hospital, and Matikwane Hospital (Mpumalanga province). Information on the patient’s age, sex, area of residence, onset of symptoms, medical history, preexisting conditions, reason for hospitalization, clinical signs, and socioeconomic data was collected using a standardized case investigation form.

### Sampling

Specimens were collected from patients that fit the case definition of AFDUC and included serum samples, whole blood, and cerebrospinal fluid (CSF) (Schubert et al., 2021). The case definition for AFDUC is that the patient has to have a fever (≥ 38°C) or history of fever in last 10 days with or without acute neurological signs or symptoms (headache, meningitis, encephalitis, acute flaccid paralysis, recent onset of Guillain–Barré syndrome, central or peripheral neurologic dysfunction), any sign of arthralgia and rash, and a negative malaria test. CSF was only taken as part of the standard of care. Serum samples were separated by centrifugation at 2,000 rpm for 15 min. Whole blood (EDTA) was used in molecular testing and red blood cells separated from plasma by centrifugation for use in serological assays for patients for whom a serum sample could not be obtained, particularly in children. All samples were aliquoted into 1.5 ml Eppendorf tubes and stored at −70°C until testing.

### Molecular Testing

Total nucleic acid was extracted and cDNA synthesized from clinical specimens from all patients enrolled in January to December 2019–2020 using the QIAamp Viral RNA Kit (Qiagen, Hilden Germany) and Expand Reverse Transcriptase (Roche, Mannheim, Germany), respectively, according to the manufacturer’s instructions (Venter et al., 2014).

A fever and meningo multiplex macro array assay developed in our lab and printed at Chipron GmBH (Berlin, Germany) was used as a differential diagnosis tool to screen for the simultaneous detection of 30 pathogens responsible for acute febrile and aseptic meningitis as previously described (Venter et al., 2014). In short, two PCR reactions using a mix of 30 primer sets labeled with biotin were performed per specimen with the Qiagen Multiplex PCR kit (Qiagen, Valencia, CA). Subsequently, the PCR products were denatured and hybridized to a custom printed microarray surface (chip), which is coated with target probes directed against each pathogen. A positive reaction was detected by addition of streptavidin-conjugated labeling enzyme (Chipron GmbH, Berlin, German) and substrate, resulting in the development of a color precipitate. The LCD array workflow (Supplementary Figure 1), pathogens targeted, and the sensitivity and specificity (Supplementary Table 1) data are provided in the supplementary data as per reference (Venter et al., 2014).

### Serological Testing

The commercial indirect IFA using Euroimmun Biochip technology was used for the serodiagnosis of IgM antibodies against SINV in sera/plasma and CSF. Samples were randomly selected from January to June in 2019 and 2020, respectively, representing a total of 197/378 of the total samples received (serum and plasma) during that time period. In the same period, a total 31/93 of all CSF specimens available from all three hospitals were selected for IgM screening for patients that had a paired serum sample. All samples were diluted 1:10 and qualitatively assessed as positive or negative. The IFA utilized commercially produced slides containing cells expressing SINV antigen that were exposed to patient serum. After incubation, fluorescein isothiocyanate-conjugated antihuman IgM was used for their detection. Fluorescence was read at a magnification of 40 × on a Nikon Eclipse 50 i microscope (Nikon, Tokyo, Japan).

### Micro Virus Neutralization Assay

Cell culture–derived SINV (19087 P4, isolated in South Africa) was titrated based on Reed and Muench (1938). The TCID50 of the virus stock was determined by preparing tenfold dilutions of virus stock in 2.0% EMEM containing FBS (Gibco, Thermo Fisher Scientific, Waltham, MA, United States) and mycozap from 10^–1^ to 10^–9^ and incubated at 37°C with 5% CO_2_ for 7 days, and CPE was observed daily. The 50% endpoint titer TCID was calculated as previously described by Reed and Muench (1938). The simplified formula used was as follows:


$$\begin{array}{l} \text{Xo}-d/\text{2}+\text{Exi/n}\\ \text{Xo = dilution at which all replicates are dead}\\ \text{d = dilution}\\ \text{Exi = sum of everything dead at the lowest concentration and every replicate thereafter}\\ n\text{ = number of replicates} \end{array}$$

Micro virus neutralization assay (micro-VNT) was used to confirm IFA positive results. Test sera was prepared in twofold dilutions (1:8; 1:16; 1:32) using 2.0% EMEM and incubated with 100TCID50 U/ml of SINV 19087 P4 in 96-well plates for 60 min at 37°C, 5.0% CO_2_. Following incubation, 80 μl of cells that were diluted to 1.5 × 104 cells per well in 2% MEM were added to each well for 4 days. The neutralization titer was determined as the reciprocal of the highest dilution of test sera at which CPE was inhibited. Specimens with a titer of ≥ 1 equivalent to a dilution of ≥ 1:8 were considered positive.

### Data Analysis

Proportion was used to characterize the demographic nature of the study population. The seropositivity rate was calculated based on the number of positive samples divided by the number of samples tested. Statistical analysis was performed using EpiInfo™ (version 7.2.0.1) using a Chi square test with a 95.0% confidence interval (CI) and odds ratios (OR) to estimate the association between clinical signs and infection (Rautenbach, 2011).

## Results

### Investigation of Acute Sindbis Virus Infection in AFDUC Patients

To identify patients acutely infected with SINV, a testing strategy involving both molecular and IgM serological testing was employed. The fever chip, a multiplex PCR assay combined with macroarray hybridization, was used to screen for 30 pathogens simultaneously, one of which was SINV (Venter et al., 2014). The second test aimed at identifying IgM antibodies specifically elicited against SINV in each patient. A total of 639 patients were tested using the fever chip (Table 1); no SINV nucleic acid was detected in any of the patients (Table 2). For IgM antibody testing, a total of 197/378 (52.0%) specimens submitted from January to June in 2019 and 2020 were randomly selected and tested. Of these 38/197 (19.3%, 95% CI: 14.0–25.5) tested positive for SINV-specific IgM antibodies using the IFA (Table 2). To confirm whether the IFA specifically identified SINV-positive patients, a SINV micro-VNT was performed on all 38 IFA-positive samples. Overall, a total of 25/38 (65.8%, 95% CI: 48.7–80.4) IFA-positive samples neutralized SINV (strain: 19087 P4), resulting in an overall IgM incidence of 12.7%. In Gauteng, 13/18 (72.2%, 95% CI: 46.5–90.3) IgM-positive specimens tested positive for neutralizing antibodies, whereas in Mpumalanga, 12/20 (60.0%, 95% CI: 36.1–80.9) were found to have neutralizing antibodies.

**TABLE 1:** Demographic characteristics and summary statistics of the study population (*N* = 639).

	Gauteng		Mpumalanga		Total
Collection year	2019	2020	2019	2020	
**Patients enrolled Jan–Dec**					
*N* patients enrolled (*n* = 639)	263 (41.2%)	162 (25.3%)	148 (23.2%)	66 (10.3%)	639 (100%)
Sample type (Jan–Dec *n* = 880)					
Whole blood	235	107	131	94	567
CSF	61	97	21	13	192
Serum	10	22	48	41	121
Total specimens	306 (34.8%)	226 (25.7%)	200 (22.7%)	148 (16.8%)	880 (100%)
**Age group (years)**					
0–5	202 (76.8%)	107 (66.0%)	74 (50.0%)	27 (40.9%)	410 (64.2%)
6–29	28 (10.6%)	14 (8.6%)	39 (26.4%)	12 (18.2%)	93 (14.6%)
30–49	27 (10.3%)	34 (21.0%)	27 (18.2%)	22 (33.3%)	110 (17.2%)
50 +	6 (2.3%)	7 (4.3%)	8 (5.4%)	5 (7.6%)	26 (4.1%)
Total	263	162	148	66	639
**Gender**					
Female	120	71	63	33	287
Male	143	91	85	33	352
^a^Sex ratios	1.19	1.28	1.35	1.00	1.23
**Statistical description**					
Min age	0	0	0	0	
Max age	64	73	81	90	
Median age	3	3	3	2	
^b^Q1	1	1	1	1	
^c^Q3	23	24	23	21	
^d^IQR	22	23	22	20	
Lower limit	−32	−34	−32	−29	
Upper limit	56	59	56	50	

*^a^Sex ratio, male/female > 1.00 → More males than females.*

*^b^Q1, Quartile 1.*

*^c^Q3, Quartile 3.*

*^d^IQR, Interquartile range.*

**TABLE 2:** Multiplex PCR, IFA (IgM), and Neutralization assay results on patients tested from 2019 to 2020.

	Gauteng		Mpumalanga		Total
	2019	2020	2019	2020	
**Enrolled Jan–June (%)**	141 (37.3)	93 (24.6)	82 (21.7)	62 (16.4)	378 (100)
**IFA tested (%)** **[95% CI]**	73 (52) [43.2–60.3]	47 (51) [40.0–61.1]	42 (51) [39.9–62.4]	35 (56 [43.3–69.0])	197 (52) [47.1–57.1]
IFA positive (%) [95% CI]	14 (19.2) [10.9–30.1]	4 (8.5) [15.6–42.6]	13 (31.0) [17.6–47.1]	7 (20) [8.4–39.9]	38 (19.3) [14.0–25.5]
**OR per site/year (%)**	(2019: 2020) 18/120 (15.0) OR = 2.55 [0.78–8.29], *p* = 0.11		(2019: 2020) 20/77 (26.0) OR = 1.79 [0.62–5.15], *p* = 0.28		1.98 [0.91–4.3], *p* < 0.05
**SINV micro-NTs**					
Percentage positivity per samples (%) [95%CI]	10/73 **(13,7)** [6.8–23.8]	3/47 **(6,4)** [1.3–17.5]	9/42 **(21.4)** [10.3–36.8]	3/35 **(8.6)** [1.8–23.1]	25/197 **(12,7)** [8.4–18.2]
**Age (%) OR** [95% CI]					
0–5	11/59 (18.6) 0.8 [0.2–3.5], *p* = 0.81	2/29 (6.9) 0.6 [0.0–4.6], *p* = 0.6	4/11 (36.4) 1.4 [0.3–6.0], *p* = 0.7	2/13 (15.4) 0.6 [0.1–3.8], *p* = 0.6	19/112 (17.0) [0.7 [0.3–1.4], *p* = 0.3
6–29	1/5 (20.0) 1.1 [0.1–10.2], *p* = 0.9	0/3 (0.0)	3/14 (21.4) 0.4 [0.1–2.2], *p* = 0.3	1/5 (20.0) 1.0 [0.1–10.7], *p* = 1	5/27 (18.5) 0.9 [0.3–2.7], *p* = 0.9
30–49	2/7 (28.6) 1.8 [0.3–10.4], *p* = 0.5	2/14 (14.3) 2.6 [0.3–20.5], *p* = 0.4	6/13 (46.2) 2.7 [0.7–10.7], *p* = 0.2	4/15 (26.7) 2.1 [0.4–11.0], *p* = 0.4	14/49 (28.6) 2.1 [0.9–4.4], ***p* ≤ 0.05**^a^
50 +	0/2 (0.0)	0/1 (0.0)	0/4 (0.0)	0/2 (0.0)	0/9 (0.0)
**Sex (%)**					
Female	10/36 (27.8) [14.2–45.2]	0/23 (0.0)	9/22 (40.9) [20.7–63.7]	5/23 (21.7) [7.5–43.7]	24/104 (23.1) [15.4–32.4]
Male	4/37 (10.81) [3.0–25.42]	4/24 (16.7) [4.7–37.4]	4/20 (20.0) [5.7–43.7]	2/12 (16.7) [2.1–48.4]	14/93 (15.1) [8.5–23.9]
**OR**	3.1 [0.9–11.3], *p* = 0.07	(0.0) *p* < 0.05	2.8 [0.7–11.1], *p* = 0.1	1.4 [0.2–8.5], *p* = 0.7	1,69 [0.82–3,51], *p* = 0,2
**Paired CSF specimen**					
Total no. of paired CSF/Sera screened	6/73 (8.2%) [3.1–17.0]	12/47 (25.5%) [13.9–40.4]	9/42 (21.4%) [10.3–36.8]	4/35 (11.4%) [3.2–26.7]	31/197 (15.7%) [10.9–21.6]
IFA (IgM) (+) in sera	2/6 **(2)** (33.3%) [4.3–77.7]	2/12 **(2)** (16.7%) [2.1–48.4]	3/9 **(2)** (33.3%) [7.5–70.1]	3/4 **(1)** (75.0%) [19.4–99.4]	10/31 **(7)** (32.3%) [16.7–51.4]
IFA (IgM) (+) in CSF	0/6 **(0)** (0.0)	1/12 **(0)** (8.3%) [0.2–38.5]	0/9 **(0)** (0.0)	1/4 **(0)** (25.0%) [0.63–80.6]	2/31 **(0)** (6.5%) [0.8–21.4]
IgM Positive in both serum and CSF	0/6 **(0)** (0.0%)	0/12 **(0)** (0.0%)	0/9 **(0)** (0.0%)	1/4 **(0)** * (25.0%)	1/31 **(0)** (3.23%) [0.1–16.7]

*Patients with paired CSF and sera tested by IgM IFA and neutralization assay positives in brackets in bold.*

*^a^P-values less than 0.05 is regarded as significant. Values in round brackets represent percentage and values in square brackets 95% CI.*

*IgM positive in sera and CSF but only neutralization positive in sera.

### Investigation of Sindbis Virus Infection in Paired Serum and Cerebrospinal Fluid Samples

As part of the standard of care, CSF samples are at times taken from AFDUC patients. Whenever possible, these samples were incorporated into the ANDEMIA study with a separate plasma or serum sample. This presented the opportunity to do a paired investigation on SINV infection from the same patient.

In 2019, of the 115 patients screened for IgM in sera/plasma, 15 patients had a paired CSF screened for IgM. Five (5/15) patients were positive for IgM in sera. In 2020, of the 82 patients tested for IgM in sera/plasma, 16 patients had a paired CSF screened for IgM of which five (5/16) of the patients were positive for IgM in sera/plasma (Table 2). Overall, 31 CSF were paired with serum samples.

One CSF specimen was IgM positive and also positive in the paired sera. The same patient was positive for neutralizing antibodies in the sera yet negative in the CSF. In addition, another CSF was IgM positive, however, was negative in the paired sera (Table 2). This same patient was negative for neutralizing antibodies in the CSF. The detection of SINV IgM in serum was higher than in CSF (OR 2.2, 95% CI 0.12–39.7, *p* > 0.6).

### Sindbis Virus-Positive Patients’ Demographics and Monthly Patterns of Sindbis Virus Infection

**FIGURE 1:**
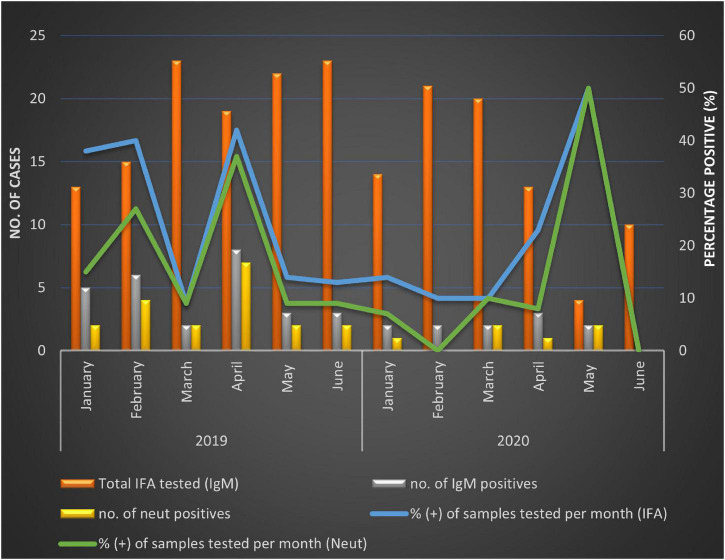
Positivity rate calculated for SINV IgM-positive specimens from patients with acute febrile and neurological disease per month between January and June in 2019 and 2020, respectively.

The majority of patients enrolled were between the ages of 0 and 5 years, 410/639 (64.2%), whereas the least number of specimens were received from persons aged 50 + years 26/639 (4.1%). However, a higher proportion of SINV IgM positivity was detected in middle-aged patients (30–49 years) (28.6%) (Table 2) but was not significantly different from younger patients (0–5 years) OR = 0.51 (95% CI 0.23–1.13), *p-*value = 0.09. Generally, SINV infections (as determined by IgM antibodies) were found in more females 24/38 (63.2%, 95% CI: 46.0–78.2) than males 14/38 (36.8%, 95% CI: 21.8–54.0). However, in 2020, men were more likely to be infected with SINV in Gauteng than females (OR = 0.0; 95% CI 0.0–1.5; *p* < 0.05). There was a higher incidence of positive cases in Mpumalanga (26.0%) than Gauteng (15.0%) (OR 1.98; 95% CI 0.91–4.3, *p* < 0.05) (Table 2). There were 27/115 (23.48%) positives in 2019 and 11/71 (13.41%) in 2020, which resulted in an overall higher positivity rate in the year 2019; 27/197 [13.7% (9.2–19.3)]. The positivity rate in 2019 accounted for 71% of the total IgM positives, OR = 1.98 (0.91–4.3), *p* = 0.08 although not statistically significant. The positivity rate was also assessed against the total number of patients that were enrolled for each month from January to June 2019 to 2020 in both sites combined to assess monthly patterns of infection. Due to the variability in sampling, percentage positivity (PP) was determined for IgM and micro-VNT results for each month in each year only (Figure 1). Peaks in positivity rate were seen in January (38%), February (40%), and April (42%) 2019 and May 2020 (50%) (Figure 1). The most significant months of detectable IgM positivity were in April OR = 2.9 [1.0–8.3] 2019, and May 2020 OR = 7.7: [1.0–61.3; *p* < 0.05].

### Clinical Signs Associated With Sindbis Virus-Infected AFDUC Patients

To determine which specific symptoms were associated with SINV disease, we compared the clinical symptoms reported by IFA anti-SINV IgM-positive AFDUC patients against SINV-negative patients. There was a positive association between three symptoms and SINV infection: headache (OR = 2.27), meningitis (OR = 2.54), and arthralgia (OR = 2.56). There was a weaker association between fatigue, dermatological signs, nausea, and HIV status. Both patients with acute flaccid paralysis were both SINV IgM– and neutralization-positive (Table 3).

**TABLE 3:** Clinical symptoms reported in AFDUC patients upon enrollment from January to June, 2019–2020.

Sign	SINV positive *N* = 38	SINV negative *N* = 159	Odds ratio	^b^*P*-value
Fatigue	29 (76.3%)	109 (68.6%)	1.48[0.65–3.35]	0.3
Headache	22 (57.9%)	60 (37.7%)	2.27[1.11–4.66]	**<0.05**
Chills	9 (23.7%)	40 (25.2%)	0.92[0.40–2.12]	0.9
Weight loss	9 (23.7%)	50 (31.5%)	0.68[0.30–1.54]	0.3
Meningitis	21 (55.3%)	52 (32.7%)	2.54[1.24–5.22]	**<0.05**
Acute flaccid paralysis	2 (5.3%)	17 (10.7%)	0.46[0.10–2.10]	0.3
Seizure	11 (28.9%)	58 (36.5%)	0.71[3.33–1.54]	0.4
Arthralgia	8 (21.1%)	15 (9.4%)	2.56[1.00–6.58]	**<0.05**
^a^Dermatological signs	7 (18.4%)	22 (13.8%)	1.41[0.55–3.58]	0.5
Fever	15 (39.5%)	70 (44.0%)	0.83[0.4–1.71]	0.6
Nausea	8 (21.1%)	18 (11.3%)	2.09[0.83–5.25]	0.11
HIV positive	12 (31.8%)	37 (23.3%)	1.52[0.70–3.31]	0.3

*The percentage (%), 95.0% Confidence interval (CI) and P-value is also indicated.*

*^a^Dermatological signs: Pruritus (itching), Skin rash, Skin patches.*

*^b^P-values less than 0.05 is regarded as significant.*

*P values < 0.05 are statistically significant.*

## Discussion

In this study, we enrolled hospitalized patients with AFDUC with or without neurological symptoms over a 2-year period from three hospitals in two provinces in South Africa. For early diagnosis in the acute phase of arbovirus infection, virus detection by RT-PCR is possible (Weaver and Smith, 2011); however, viremia during SINV infection lasts only for a few days, and low-level acute infections may be missed. We utilized a differential diagnostic PCR-based assay that can detect 30 causes of fever and/or neurological signs, including SINV, that was previously shown to be of high sensitivity and specificity (Venter et al., 2014; Guillebaud et al., 2018) to screen all cases across the 2 years, and cases submitted during the arbovirus season (late summer and autumn months), January to the end of June, were also screened for SINV-specific IgM antibodies. The arbovirus season was previously defined through arbovirus-positive cases detected in humans by the National Institute for Communicable Disease (NICD) reference laboratory and in fever and neurological disease surveillance in horses by the ZARV program at the University of Pretoria (van Niekerk et al., 2015).

Although no patients tested positive for SINV RNA, SINV-specific IgM antibodies were detected using a commercial SINV-specific IFA as a serological assay. This can be explained by previous observations that indicate that the end of viremia coincides with the first appearance of IgM antibodies (Weaver and Paessler, 2009). Therefore, hospitalized patients may have already passed the viremic stage, justifying our identification of only IgM-positive patients. All IgM-positive specimens were confirmed using the microneutralization assay to rule out cross-reactions to other alphaviruses. Our results indicate that the incidence of IgM antibodies against SINV in the cohort of patients with fever of unknown origin in 2019–2020 was 19.0%, whereas 12.7% had SINV-neutralizing antibodies. The findings were similar to the previous study conducted by Storm et al. (2014a) in which the detection rate of SINV IgM was 10% in 2010 in arbovirus diagnostic submissions to the NICD (Storm et al., 2014a). Diagnostic submission to the NICD reflects mainly milder cases with typical arbovirus symptoms of rash, fever, and arthralgia. The current study is unique in that we did active surveillance, investigating hospitalized patients with fever with or without neurological signs. The appearance of IgM antibodies begins approximately 1 week postinfection, whereas IgG antibodies begin at 2 weeks. The IgM response consists of both neutralizing and non-neutralizing antibodies (Weaver and Paessler, 2009). This could explain the discrepancy between IgM and neutralization in our data. Furthermore, the potential for cross-reactivity in the IFA cannot be ruled out (Chua et al., 2017; Fischer et al., 2020). Cross-reactivity can be possible with other alphaviruses, such as CHIKV, MIDV, or ONNV (Burt et al., 2012; Van Niekerk et al., 2015; Chua et al., 2017; Henss et al., 2019; Lattwein et al., 2019). In SA, MIDV is endemic, whereas CHIV is rare and ONNV is not detected (Venter, 2018). This suggests that other alphaviruses may also be present in these regions and should be investigated further. Neutralization assays, therefore, increase the reliability in ruling out false positives due to cross-reactivity.

There were more patients enrolled in 2019 (64.3% of total) than in 2020 (35.7%). There was a significant decrease of almost 50% in enrollment numbers in 2020. The reduced number of enrollments between April and June 2020 was related to lockdown regulations in South Africa due to the COVID-19 pandemic. Positivity rate fluctuated between the months; however, April 2019 and May 2020 had statistically significant associations with SINV infection. These results are similar to earlier findings that suggest SINV epidemics occur more frequently following high-rainfall summer months, which favors mosquito breeding (Rautenbach, 2011) in both Gauteng and Mpumalanga (Jupp and Nk, 1986; Storm et al., 2014a).

The majority of patients were enrolled from Gauteng, 425/639 (66.5%) over Mpumalanga, 214/639 (33.5%) (Table 1). Overall, a higher IgM positivity rate and a statistically significant association with SINV-positive tests was, however, seen in Mpumalanga with a percentage positivity of 31 and 20% in 2019 and 2020, respectively (Table 2), respectively. The hospitals in Mpumalanga province are located in rural sites with patients having an outdoor lifestyle, being in closer contact with wildlife and/or livestock and the climatic and biodiversity conditions possibly favoring breeding of mosquitoes.

The average age of persons infected with SINV was the middle aged (30–49 years). The study agrees with previous results seen in Storm et al. (2014a) in that the risk of acquiring SINV increased linearly with age ≤ 5 years (17.0%), 6–29 years (18.5%), and 30–49 years (28.6%) (Table 3). The high level of SINV detections in this study is surprising, suggesting it may be missed as a cause of AFDUC in children in SA, possibly due to several other causes associated with rash and fever in the younger age group. The findings were comparable to results found by Kurkela et al that show the average age of persons infected with SINV was 41 years (Kurkela et al., 2005, 2008). This could suggest that adults have a higher risk for SINV infection. There was, however, no detection of SINV in patients over 50 years of age although this may be a result of a very low enrollment number in that age group.

SINV has been identified in horses with febrile and neurological signs, including fatal cases that had lesions of meningoencephalitis on postmortem brain samples that tested RT-PCR positive in SA (Van Niekerk et al., 2015). CSF specimens taken as part of standard of care were screened for SINV-specific IgM with the IFA. The detection rate was 2/31 (6.45%, 95% CI: 0.79–21.42). One CSF specimen (ZRU1329/20) had SINV IgM-positive paired plasma that was also confirmed positive with a SINV micro-NT assay. The second CSF specimen that tested positive for SINV IgM on the IFA had a paired plasma sample that tested negative for anti-SINV IgM, and this may suggest that this was a false positive. IFA assays may also be less effective on CSF. The negative neutralization results for these CSF specimens may suggest that other alphaviruses may be responsible for these infections or that it is SINV infection with neurological signs, but the IgM did not cross the blood–brain barrier. The observed neurological sign may be immune-induced rather than a direct effect of the virus. The analysis between paired CSF and sera was undertaken to determine if serum positivity correlated with CSF positivity in patients with neurological signs, suggesting that these patients had a blood–brain barrier breach and also if CSF could substitute for sera as a diagnostic specimen for acute SINV infection with this IFA assay. Despite an OR = 2.22 (95% CI 0.12–39.7), indicating an association of detecting anti-SINV IgM antibodies in both sample types using the IFA, the large CI suggested more paired samples are required to conclude if CSF is a reliable indicator of IgM positivity.

Historically, SINV is shown to cause significant morbidity with the most common feature being arthritis, which may persist for years (Calisher and Monath, 1988; van Niekerk et al., 2015). The most frequently observed symptom associated with SINV infections is fatigue 29/38 (76.3%), chills 22/38 (57.9%), and meningitis 21/38 (55.3%) in the current study. Acute flaccid paralysis is reported in two of the patients that were positive for anti-SINV IgM and neutralizing antibodies. One of the patients was a middle-aged (34 years) patient who was also HIV positive. The second patient was an HIV-negative infant (1 year old), and upon a 1 month follow up, this case was reported as a fatality. Overall, two fatalities were reported; the second fatality was a 10-year-old child who was positive for SINV IgM, but the case could not be confirmed with neutralization assays. Symptoms that were significantly associated with a positive SINV test were headache, meningitis, and arthralgia. Thirty-one percent of SINV-infected patients (12/38) were HIV positive, and 9/12 (75%) of them were middle-aged (30–49 years) patients. All of these patients presented with meningitis and a headache. The high HIV prevalence may potentially contribute to this high incidence of SINV in hospitalized cases in South Africa, in particular, in adults although it was not statistically significantly associated with SINV infections. The majority of SINV-positive patients were not HIV positive and in the younger age group. The incidence of SINV in AFDUC in children < 5 warrants further investigation and may be missed when confused with other childhood diseases in Africa such as parvovirus B19 infection (Reid et al., 1985), varicella, measles, and rubella (Kurkela et al., 2005).

The limitation of the study is that IFA is subject to individual interpretation, which may result in false positives being reported (Calisher and Monath, 1988). A subset of the samples collected were tested because an IFA can only test a limited number of samples. Furthermore, 93 CSF specimens were collected from January to June 2019–2020; however, 31/93 (33.3% [95%CI 23.9–43.9]) paired CSF specimens were tested due to limited volume of CSF received and priority to molecular testing. The IFA IgM antibody and neutralization assays were performed on a single sample, and a limitation of the study is that there was no possibility of a follow-up sample. There is evidence provided by Laine et al. (2000) of SINV IgM antibodies persisting as long as 2.5 years after SINV fever. Therefore, it is possible that a proportion of these infections may not have been acute. A significant titer increase in a follow-up sample taken at a correct time interval would be suitable for indicating an acute infection. The other limitations of the study are primarily the relatively low numbers of samples under analysis and the timespan over which the samples were collected. Other arboviruses that are closely related to SINV may have been responsible for the symptoms of the patients. However, these potential weaknesses in the investigation can be addressed in future, wider-ranging studies.

To conclude, SINV contributed significantly to AFDUC cases in hospitalized patients during the late summer and autumn months in SA. Despite the historical SINV outbreaks and annual sporadic cases reported in SA, limited epidemiological data on hospitalizations in SA or other African countries existed prior to this study. The association with neurological signs reported here suggest the severity of the disease may be underestimated and needs further investigation. It is, therefore, important for medical health practitioners to be aware and familiar with zoonotic infections, how they present themselves as well as the potential for outbreaks. Because there is no known treatment, cure, or vaccine for most of the arboviruses, such as SINV, preventative measures, such as vector control, can be introduced.

## Data Availability Statement

The original contributions presented in the study are included in the article/Supplementary Material, further inquiries can be directed to the corresponding author/s.

## Ethics Statement

Ethical approval to conduct this study was granted by the University of Pretoria, Faculty of Health Sciences Research Ethics Committee Protocols 100/2017 (ANDEMIA study) and 101/2017 (National Health Laboratory Services, Virology Laboratory), 842/2019 and HUM017/1119 (MSc project ethics approval). Hospital approvals were obtained from Kalafong Hospital KPTH 33/2017 and the Department of Health Mpumalanga Province. Written informed consent to participate in this study was provided by the participants’ legal guardian/next of kin.

## Author Contributions

MV: conceptualization. KM, AM, and MV: methodology, formal analysis, and writing—review and editing. KM, AM, CY, and MV: investigation. KM: writing—original draft preparation. All authors have read and agreed to the published version of the manuscript.

## Conflict of Interest

The authors declare that the research was conducted in the absence of any commercial or financial relationships that could be construed as a potential conflict of interest.

## Publisher’s Note

All claims expressed in this article are solely those of the authors and do not necessarily represent those of their affiliated organizations, or those of the publisher, the editors and the reviewers. Any product that may be evaluated in this article, or claim that may be made by its manufacturer, is not guaranteed or endorsed by the publisher.

## Footnotes

1
www.nicd.ac.za
