# Supplementary material for: Incidence of Sindbis Virus in Hospitalized Patients With Acute Fevers of Unknown Cause in South Africa, 2019–2020
Source: Front Microbiol. 2022 Feb 7;12:798810. doi: 10.3389/fmicb.2021.798810 (PMC8860305; doi:10.3389/fmicb.2021.798810)
Supplement: Supplementary file 1 [file Data_Sheet_1.PDF]

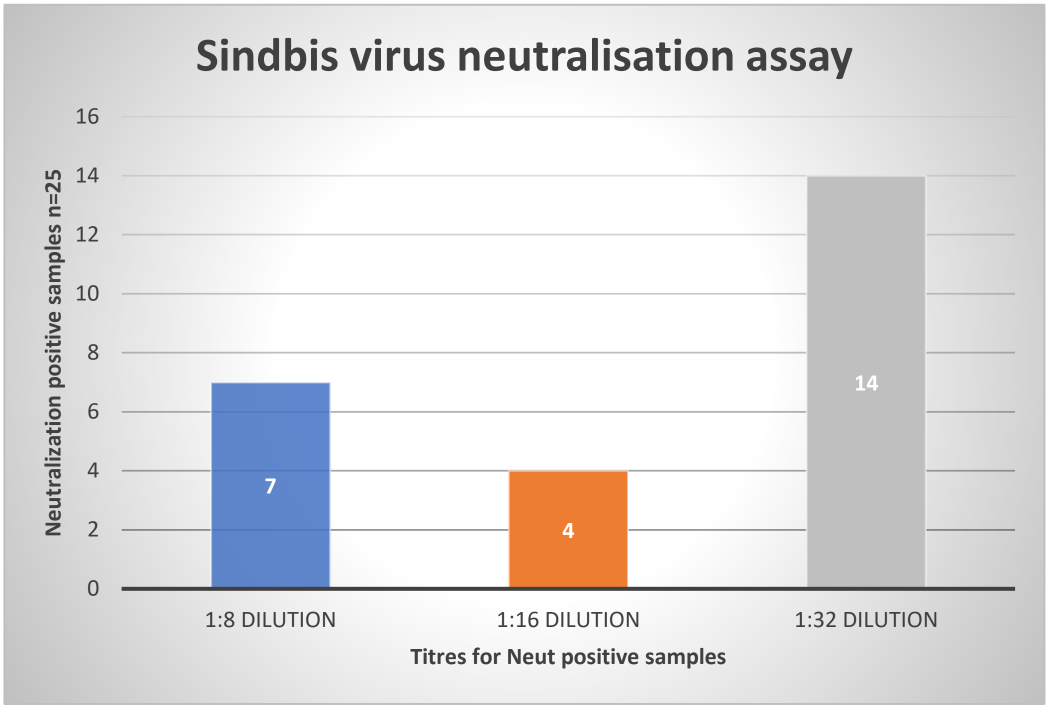

Supplementary Figure S 1: Data representing the end-point titers of the samples tested in the neutralization antibody assay.

Table S 1: Human IFA and neutralization assay results

Samples	IFA result	1:8	1:16	1:32
ZRUA0297/19	+	+	-	-
ZRUA0301/19	+	-	-	-
ZRUA0311/19	+	-	-	-
ZRUA0312/19	+	+	+	-
ZRUA0313/19	+	-	-	-
ZRUA0330/19	+	+	-	-
ZRUA0332/19	+	-	-	-
ZRUA0357/19	+	+	-	-
ZRUA0426/19	+	+	+	+
ZRUA0428/19	+	-	-	
ZRUA0431/19	+	+	-	-
ZRUA0458/19	+	+	+	-
ZRUA0469/19	+	+	-	-
ZRUA0495/19	+	+	+	+
ZRUA0496/19	+	+	+	+
ZRUA0500/19	+	+	+	+
ZRUA0525/19	+	+	+	+
ZRUA0530/19	+	+	+	+
ZRUA0543/19	+	+	+	-
ZRUA0546/19	+	+	+	+
ZRUA0549/19	+	-	-	-
ZRUA0573/19	+	+	-	-
ZRUA0581/19	+	-	-	-
ZRUA0607/19	+	+	+	+
ZRUA0635/19	+	+	+	+
ZRUA0665/19	+	+	+	+
ZRUA0681/19	+	-	-	-
ZRUA1117/20	+	+	+	-
ZRUA1147/20	+	-	-	-
ZRUA1160/20	+	-	-	-
ZRUA1203/20	+	-	-	-
ZRUA1226/20	+	+	+	+
ZRUA1267/20	+	+	+	+
ZRUA1329/20	+	+	-	-
ZRUA1338/20	+	-	-	-
ZRUA1361/20	+	-	-	-
ZRUA1380/20	+	+	+	+
ZRUA1382/20	+	+	+	+
Total	38	7/25	4/25	14/25

Table S 2: Pathogens targeted by the array, indicating the detection limit for each target.

Chipron LCD-array Pathogen	Assay sensitivity (plasmid copies)
West Nile virus	1.46
Rift Valley Fever virus	1.43
Chikungunya virus	1.47
Sindbis virus	1.45
Rubella virus	14.0
Crimean Congo Haemorrhagic Fever virus	10.4
Cytomegalovirus	14.0
Measles virus	13.5
Mumps virus	13.9
Herpes simplex virus 1	1.37
Herpes simplex virus 2	1.36
Varicella Zoster virus	14.3
Rabies virus	1.36
Epstein–Barr virus	1.45
JC virus	14.5
Enterovirus	1.34
Dengue virus	1.44
Rickettsia spp.	1.47
Borrelia burgdorferi/garinii	14.1
Brucella spp.	13.6
Adenovirus	1.41
Coxiella burnetti	1.39
Leptospira spp.	1.39
Mycobacterium tuberculosis	13.9
Ehrlichia spp.	1.40
Neisseria meningitidis	1.47
Plasmodium falciparum	1.48
Flavivirus genus	1.41
Hepatitis-A virus	14.8
Hepatitis-B virus	1.42

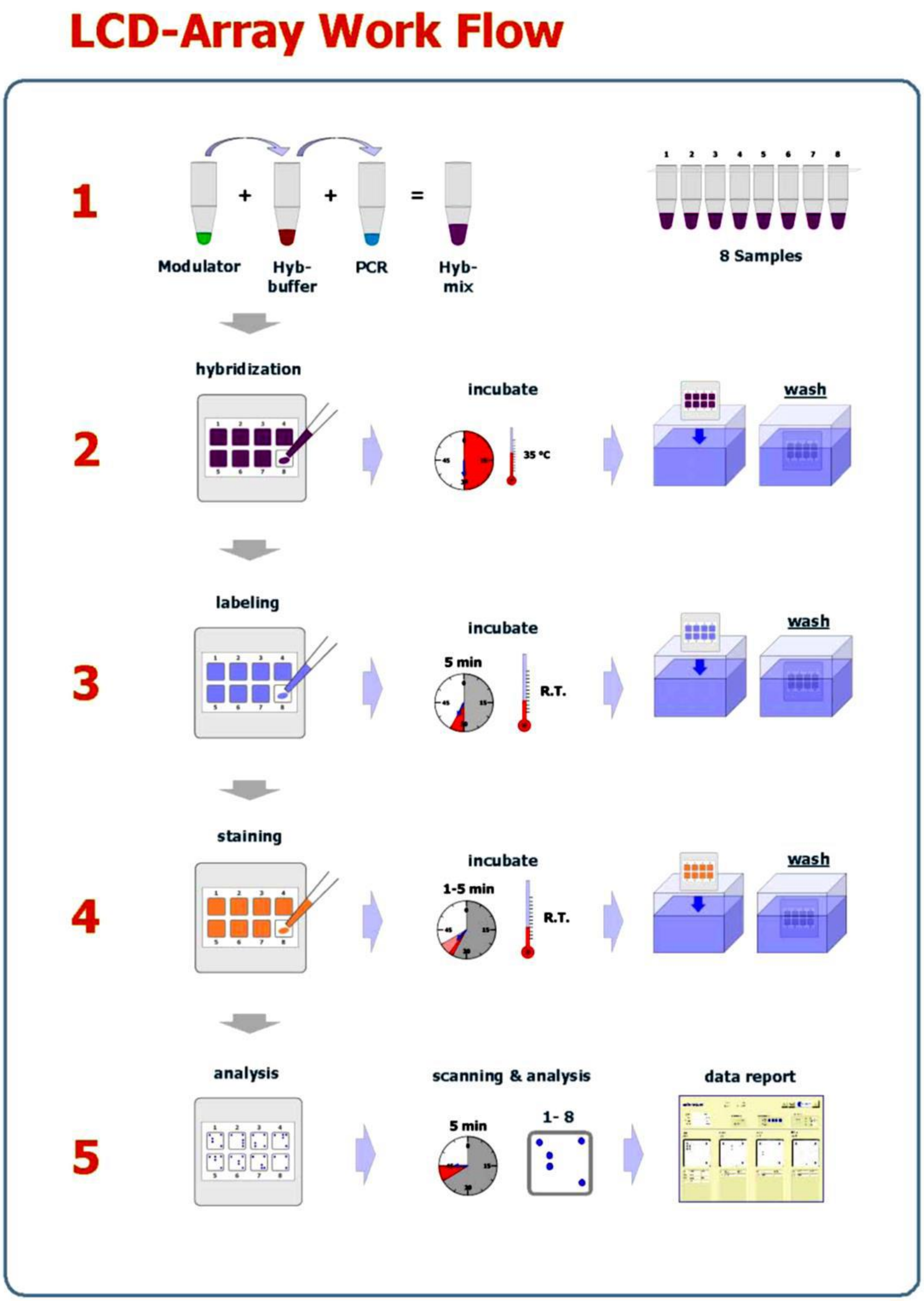

Figure S 2: LCD array workflow summarizing the multiplex PCR using the Chipron-LCD array
